# Veterinary Department of the University of California

**Published:** 1896-09

**Authors:** 


### VETERINARY DEPARTMENT OF THE UNIVERSITY OF CALIFORNIA.

CHANGES in the Board of Regents bring this year to the support of the university system of this State W. T. Jetard, Lieutenant-Governor, in place of Spencer G. Millard. Ernest A. Denicke succeeds A. S. Hallidie; Columbus Bartlett and George J. Ainsworth succeed J. B. Reinstein and John E. Budd. F. F. Knorp has been added as assistant professor of physiology and histology, and D. F. Reeves as assistant professor of chemistry. K. O. Steers, V.S., succeeds A. A. Cunningham, F.C.S., as secretary.

